# Sparking Nano-Metals on a Surface of Polyethylene Terephthalate and Its Application: Anti-Coronavirus and Anti-Fogging Properties

**DOI:** 10.3390/ijms231810541

**Published:** 2022-09-11

**Authors:** Kittisak Jantanasakulwong, Sarinthip Thanakkasaranee, Phisit Seesuriyachan, Pisith Singjai, Aphisit Saenjaiban, Siriphan Photphroet, Kanticha Pratinthong, Yuthana Phimolsiripol, Noppol Leksawasdi, Thanongsak Chaiyaso, Sarana Rose Sommano, Pensak Jantrawut, Siriwadee Chomdej, Suwit Chotinan, Francisco J. Barba, Joe M. Regenstein, Alissara Reungsang, Pornchai Rachtanapun

**Affiliations:** 1 Cluster of Agro Bio-Circular-Green Industry, School of Agro-Industry, Faculty of Agro-Industry, Chiang Mai University, Chiang Mai 50100, Thailand; 2 Department of Physics and Materials Science, Faculty of Science, Chiang Mai University, Chiang Mai 50200, Thailand; 3 Doctor of Philosophy Program in Nanoscience and Nanotechnology (International Program/Interdisciplinary), Faculty of Science, Chiang Mai University, Chiang Mai 50200, Thailand; 4 Department of Chemistry, Faculty of Science, Chiang Mai University, Chiang Mai 50200, Thailand; 5 Plant Bioactive Compound Laboratory (BAC), Department of Plant and Soil Sciences, Faculty of Agriculture, Chiang Mai University, Chiang Mai 50200, Thailand; 6 Faculty of Pharmacy, Chiang Mai University, Chiang Mai 50200, Thailand; 7 Department of Biology, Faculty of Science, Chiang Mai University, Chiang Mai 50200, Thailand; 8 Faculty of Veterinary Medicine, Chiang Mai University, Chiang Mai 50100, Thailand; 9 Department of Preventive Medicine and Public Health, Food Science, Toxicology and Forensic Medicine, Faculty of Pharmacy, University of Valencia, 46100 Valencia, Spain; 10 Department of Food Science, Cornell University, Ithaca, NY 14853-7201, USA; 11 Department of Biotechnology, Faculty of Technology, Khon Kaen University, Khon Kaen 40002, Thailand

**Keywords:** sparking process, polyethylene terephthalate, anti-virus, nanoparticle, coronavirus

## Abstract

The nano-metal-treated PET films with anti-virus and anti-fogging ability were developed using sparking nano-metal particles of Ag, Zn, and Ti wires on polyethylene terephthalate (PET) films. Ag nanoparticles were detected on the PET surface, while a continuous aggregate morphology was observed with Zn and Ti sparking. The color of the Ag-PET films changed to brown with increasing repeat sparking times, but not with the Zn-PET and Ti-PET films. The water contact angle of the nano-metal-treated PET films decreased with increasing repeat sparking times. The RT-PCR anti-virus test confirmed the high anti-virus efficiency of the nano-metal-treated PET films due to the fine particle distribution, high polarity, and binding of the nano-metal ions to the coronavirus, which was destroyed by heat after UV irradiation. A highly transparent, anti-fogging, and anti-virus face shield was prepared using the Zn-PET film. Sparking was an effective technique to prepare the alternative anti-virus and anti-fogging films for medical biomaterial applications because of their low cost, convenience, and fast processing.

## 1. Introduction

Nanotechnology is a rapidly expanding discipline, which refers to the development and use of structures, devices, and systems with unique features and functionalities because of their small size and the capacity to control or manipulate matter at the nanoscale level [1,2]. Nanoparticles (NP) have been used in various applications such as automotive, chemical engineering, medicine, cosmetic, and food due to their dimension being <100 nm, high surface area to volume ratio, and well-specific properties [3,4].

Metal NP (i.e., Ag [5], Zn [6], and Ti [7]) and metal oxide NP (i.e., ZnO [8], TiO_2_ [9], and CaO [10]) have been continuously studied by researchers because of their important properties (e.g., antimicrobial properties [11], UV blocking [12], and anti-inflammatory properties [13]), which have been used in biomedical [12], food packaging [14], and cosmetic applications [15]. In addition, Ag [5,16,17], Zn, [6] and Ti [7,18] have been investigated as inhibitors of coronavirus by binding with the virus, absorbing UV radiation, conversion of the UV/light energy into heat energy, and destroying viruses [19,20]. This mechanism usually occurs with near-infrared radiation adsorption, which is an important factor for antiviral activity of such metal NP [21]. Ag NP and Ti NP interact and destroy the virus envelope [22,23]; Ag NP, Au NP, and Cu NP destroy the viral protein of the virus by physical damage [24,25,26]; and Ti NP binds and destroys viral capsid protein through a photocatalytic effect [27,28]. In the case of the metal oxide form (e.g., TiO_2_), with UV radiation, surface proteins of the viruses are oxidized through contact with the surface of TiO_2_ and/or ROS are generated, which are the dominant factors of their antiviral mechanism, leading to degradation of the envelop/capsid, nucleic acid leakage, and virus degradation/virus inactivation [29,30].

Despite its medical application, few studies have been carried out on metal NP as an antiviral material. For example, the coronavirus disease spreads rapidly and causes fever, dry cough, and fatigue. Moreover, the coronavirus 2019 (COVID-19) pandemic showed a high rate of infection of new cases and deaths in China, the United States, and India [31], among others, and the virus is still spreading in every country. Therefore, personal protective equipment (PPE) is required for COVID-19 protection, such as gloves, masks, face shields, and shoe covers, while respirators are required in emergency clinics. However, coronavirus still remains on surfaces and can infect humans. Thus, development of an alternative antiviral material based on metal NP and polymeric films using the facile method was studied.

Various methods have been used to generate and deposit NP on thin films including sputtering [32], chemical processes [33], sol-gel transformations [34], electrodeposition, [35] laser radiation, [36] and spray pyrolysis [37]. However, these processes use toxic chemicals, require many steps, take a long time, and are expensive. Therefore, several studies attempt to develop and design effective methods that can address such drawbacks. The sparking process is an alternative and effective method because it is simple, low-cost, and rapid, as well as using non-toxic starting materials (i.e., metal wires) to generate metal NP and change the surface of materials [38,39,40]. This process can deposit NP on the surface of a thin film without needing a vacuum system. Potentially, to help prevent COVID-19 infections, using the sparking process to develop antiviral films with metal NP deposits and determine their antiviral properties might be beneficial.

Therefore, the objective was to develop anti-fogging and antiviral films using a sparking process. Ag, Zn, and Ti wires were used as substrates for deposition of NP on a polyethylene terephthalate (PET) film surface. The effects of metal ion and repeated sparking on the morphology, color, contact angle, and anti-virus properties were investigated.

**Figure 1:**
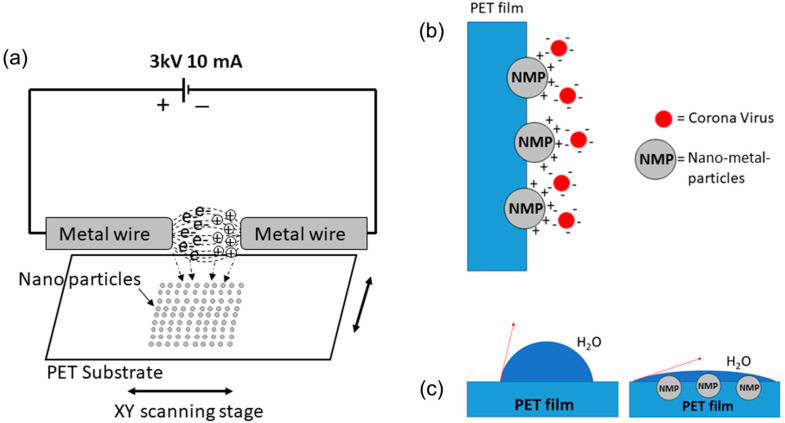
Schematic illustrations of the nano-metal-coated PET surface using the sparking technique (**a**) schematic diagram of the sparking apparatus for PET film, (**b**) schematic of the nano-metal particles deposited on the PET surface and coronavirus binding, (**c**) schematic of the reduced surface tension with the deposited NP.

The sparking process was used to generate NP on the surface of a PET film. Three different metal wires were used, and their NPs were embedded on the surface of the PET film (Figure 1a). The polarity of the NP was thought to aid in the binding to coronavirus, and the natural UV light photocatalytically heated the NP, thereby inactivating the virus [41] (Figure 1b). A low surface tension because of the deposited NP was also expected due to the polarity of NP (Figure 1c), which also might provide anti-fogging properties.

## 2. Results and Discussion

### 2.1. Morphology of the Nano-Metal Treated PET Film Surface

**Figure 2:**
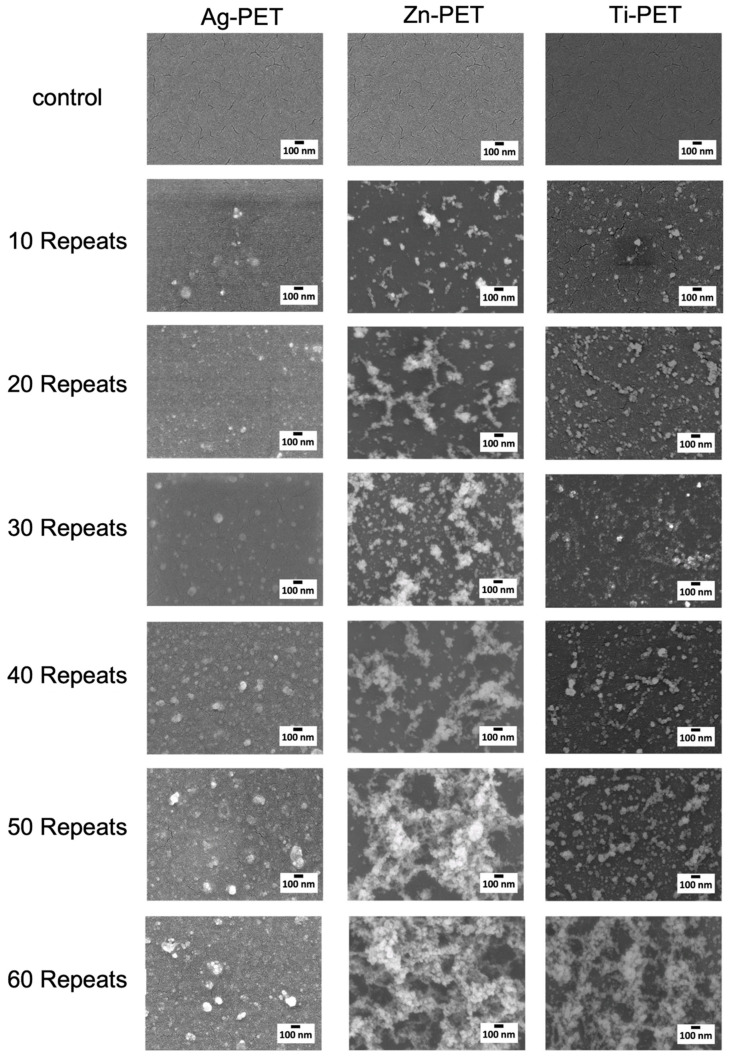
Scanning electron microscope (SEM) images of the Ag-PET, Zn-PET, and Ti-PET films repeated 0–60 times.

The SEM images of the nano-metal-treated PET film surfaces with different numbers of sparking times are shown in Figure 2. The untreated PET film shows smooth surfaces and some cracks attributing to the relatively high-energy electrons during the SEM investigation. NPs of Ag (20–60 nm), Zn (10–20 nm), and Ti (10–20 nm) were observed on the sparked PET surface, and the size of the NP was unchanged with different numbers of sparking although the amount of NP increased. Agglomeration of Zn NP was observed 20 to 60 times. Small particles of Ti NP were observed 10 to 40 times, which became aggregated thereafter.

**Figure 3:**
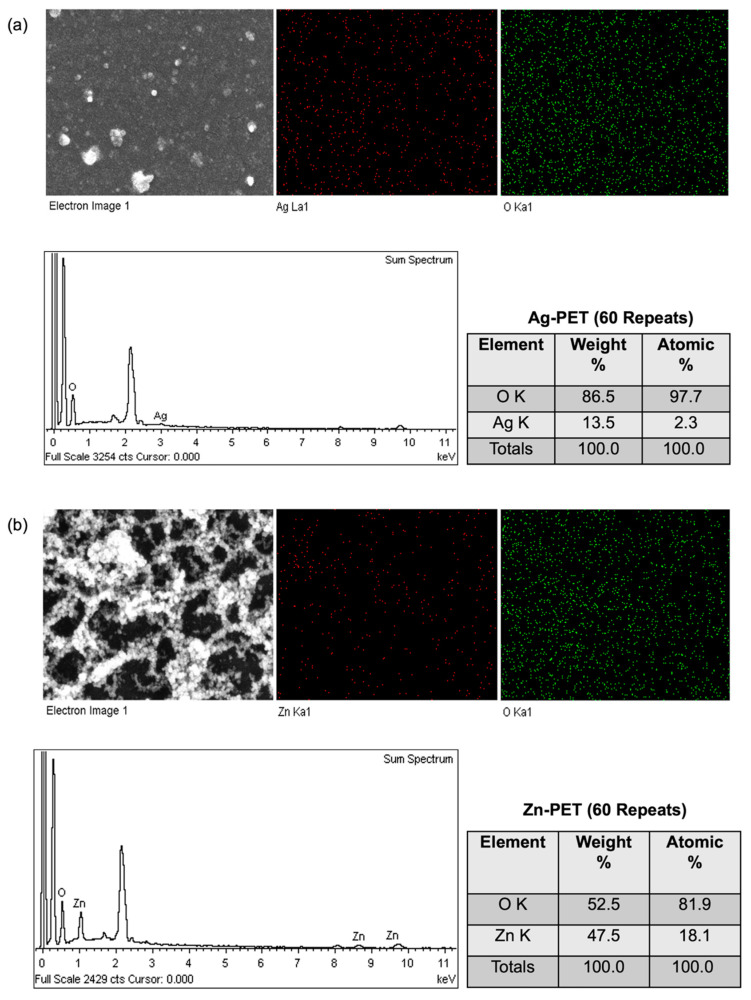

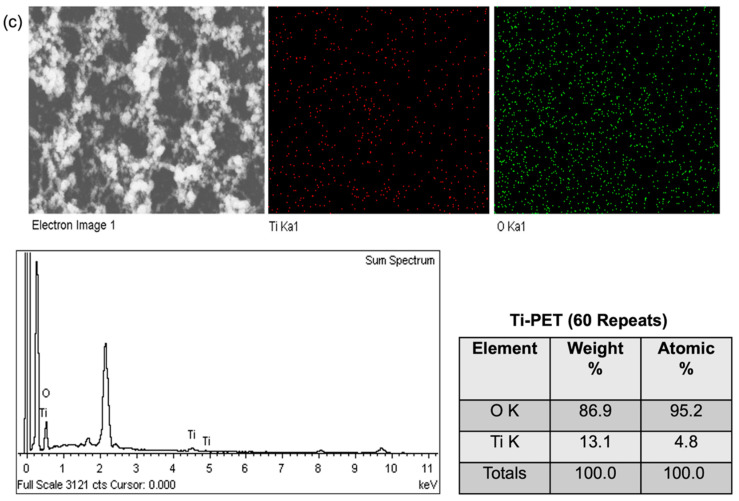
Field emission scanning electron microscope (FESEM) (magnification: 50,000×) with X–ray element mapping of the Ag-PET (**a**), Zn-PET (**b**), and Ti-PET (**c**) films treated 60 times.

The chemical elements of the treated PET films were observed using EDS with FE-SEM. Free Ag, Zn, and Ti were 2.3, 18.1, and 4.8%, respectively (Figure 3), and the oxygen (O) was 97.7 (Ag-PET), 81.9 (Zn-PET), and 95.2% (Ti-PET), when using the respective metal oxide (AgO, ZnO, and ZnO). The strong peaks at 0.3 and 3 keV were C and Au, corresponding to the PET and the gold sputtering of samples prior to SEM-EDX analysis. The SEM images, strong FESEM-EDX signal, and the elemental analysis confirmed the fine metal-oxide NP distribution on the PET film surface.

**Figure 4:**
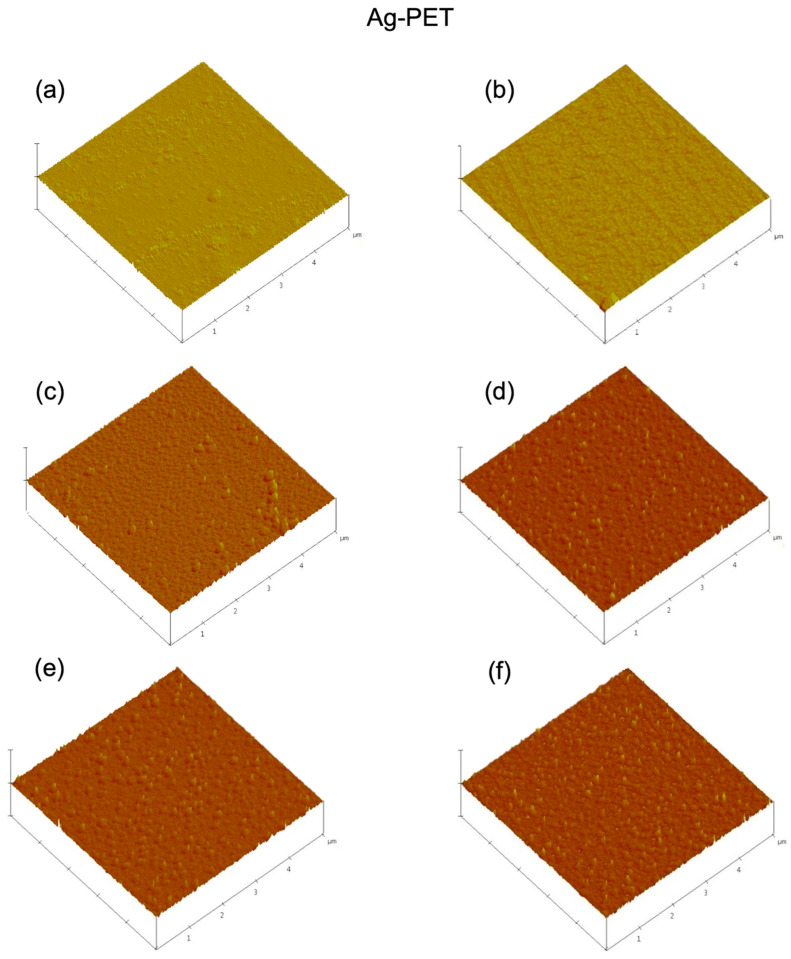

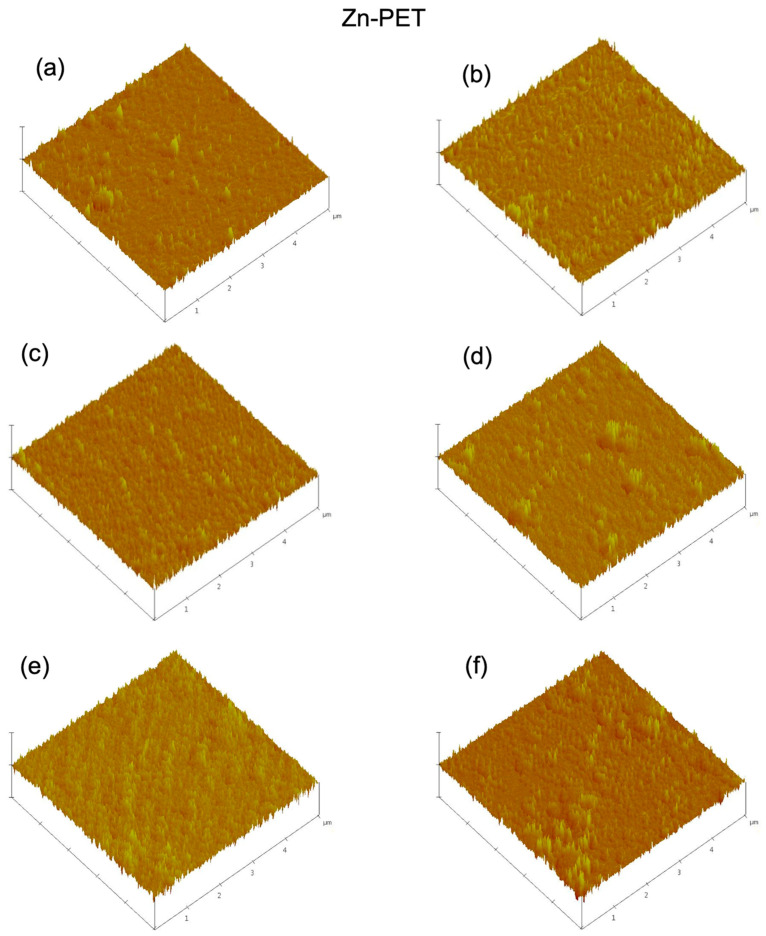

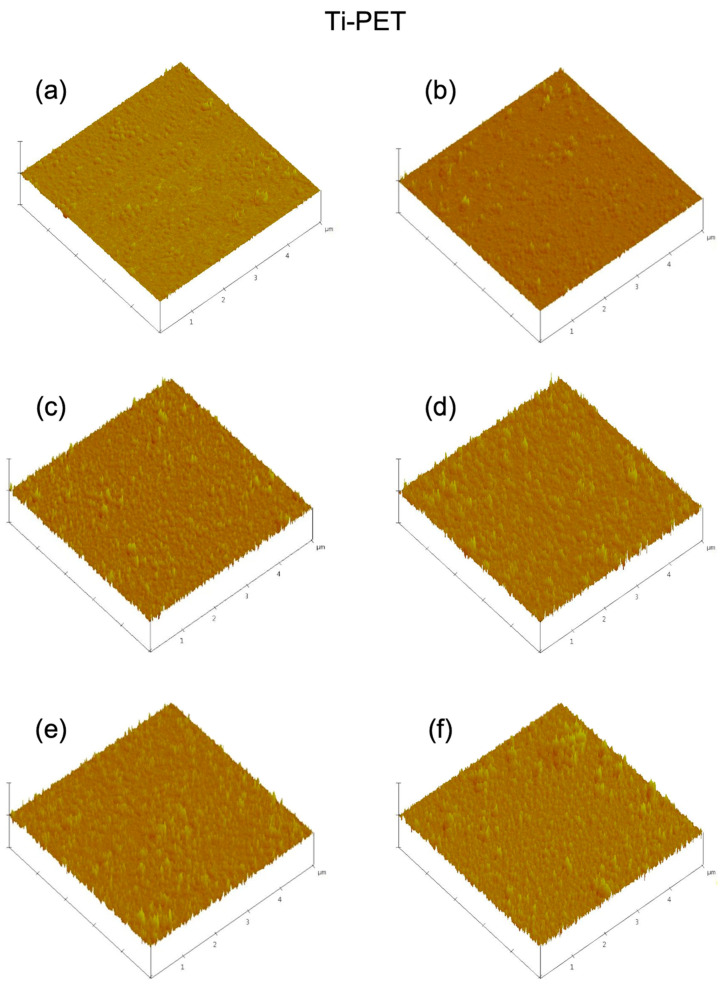
Atomic force microscopy (AFM) images of the Ag-PET, Zn-PET, and Ti-PET films treated (**a**) 10, (**b**) 20, (**c**) 30, (**d**) 40, (**e**) 50, and (**f**) 60 times.

AFM 3D roughness surface images (Figure 4 and Table 1) confirmed an increase in aggregations of NP and surface roughness with repeat sparking times. The agglomeration of Zn and Ti NP was due to the high surface energy of the small NP (10–20 nm), the effect of the ions, and the energetic NP states (such as excited nitrogen) that occurred during sparking [42]. For example, the higher energetic NP states of sparked Zn and Ti at 60 repeats were consistent with the lower oxide content compared to the Ag reported above. The larger initial NP size distribution of Ag particles was probably due to the higher thermal and electrical conductivity of Ag [43], and lower energetic NP states [42]. Large continuous aggregates of Zn and Ti NP when sparked has been reported [39].

### 2.2. Colors and Visual Images of the Nano-Metal Treated PET Films

**Figure 5:**
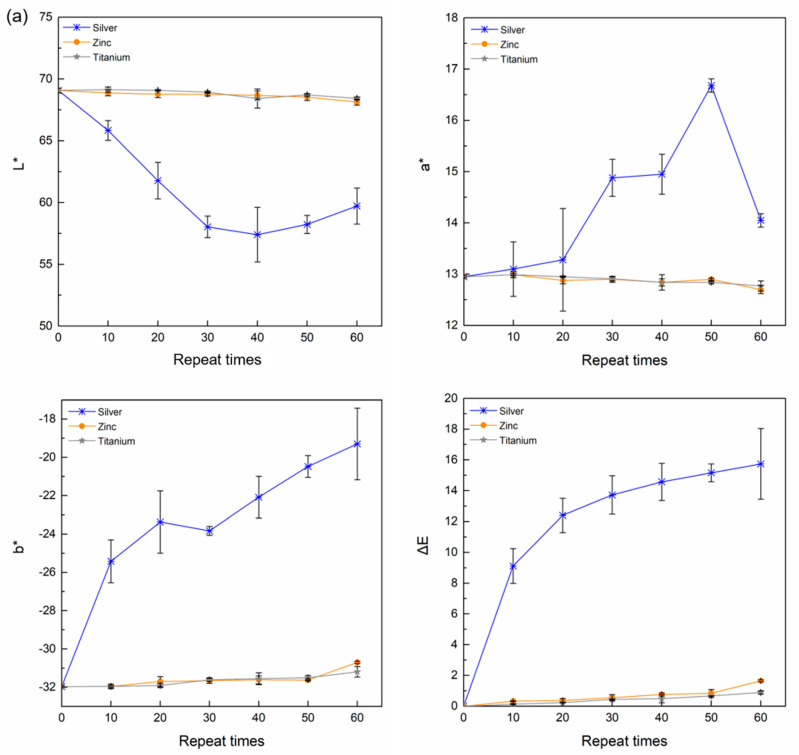

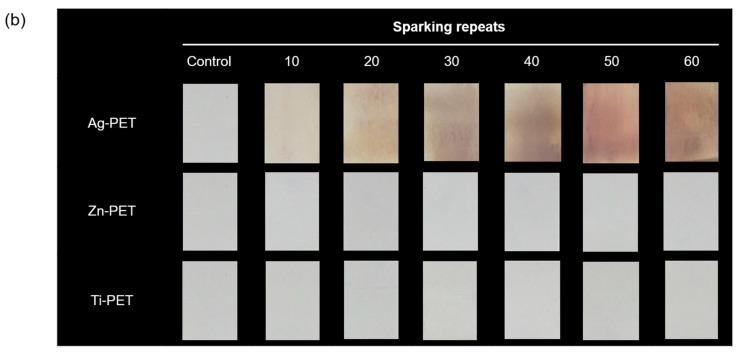
L*, a*, b* values (**a**) and visual images (**b**) of the Ag-PET, Zn-PET, and Ti-PET films treated 0–60 times.

The PET films were sparked with Ag, Zn, and Ti and stored at 25 °C and 54% RH for 24 h prior to color measurement. L* is a measure of lightness, a* is the chroma from green to red, b* is the chroma from blue to yellow, and ΔE is the total color difference [44]. Figure 5a shows the L*, a*, b*, and ΔE of the untreated and treated PET films with 0–60 repeats. The L* of the Ag-treated samples decreased at 30–60 repeats, while the a*, b*, and ΔE of the Ag-treated samples increased with repeats. The L* and a* of the Zn and Ti samples slightly decreased, while b* and ΔE showed a non-significant increasing trend. The Ag samples showed a decreased brightness (low L*) because the high temperature during sparking induced a color change [45], nucleation of Ag+ ions, and smaller Ag particles [46]. The decrease in L* with temperature has been previously reported [47]. High a* and b* led to a color change of the film to red and yellow tones. The ΔE of the Ag samples confirmed a significant color variability, while the Zn and Ti samples showed an insignificant change. Visual images of the treated samples are shown in Figure 5b. The color change in polymers due to the addition of nano-Ag particles with high temperature and UV light exposure has been previously reported [45,47].

### 2.3. Surface Tension of the Nano-Metal Treated PET Films

**Figure 6:**
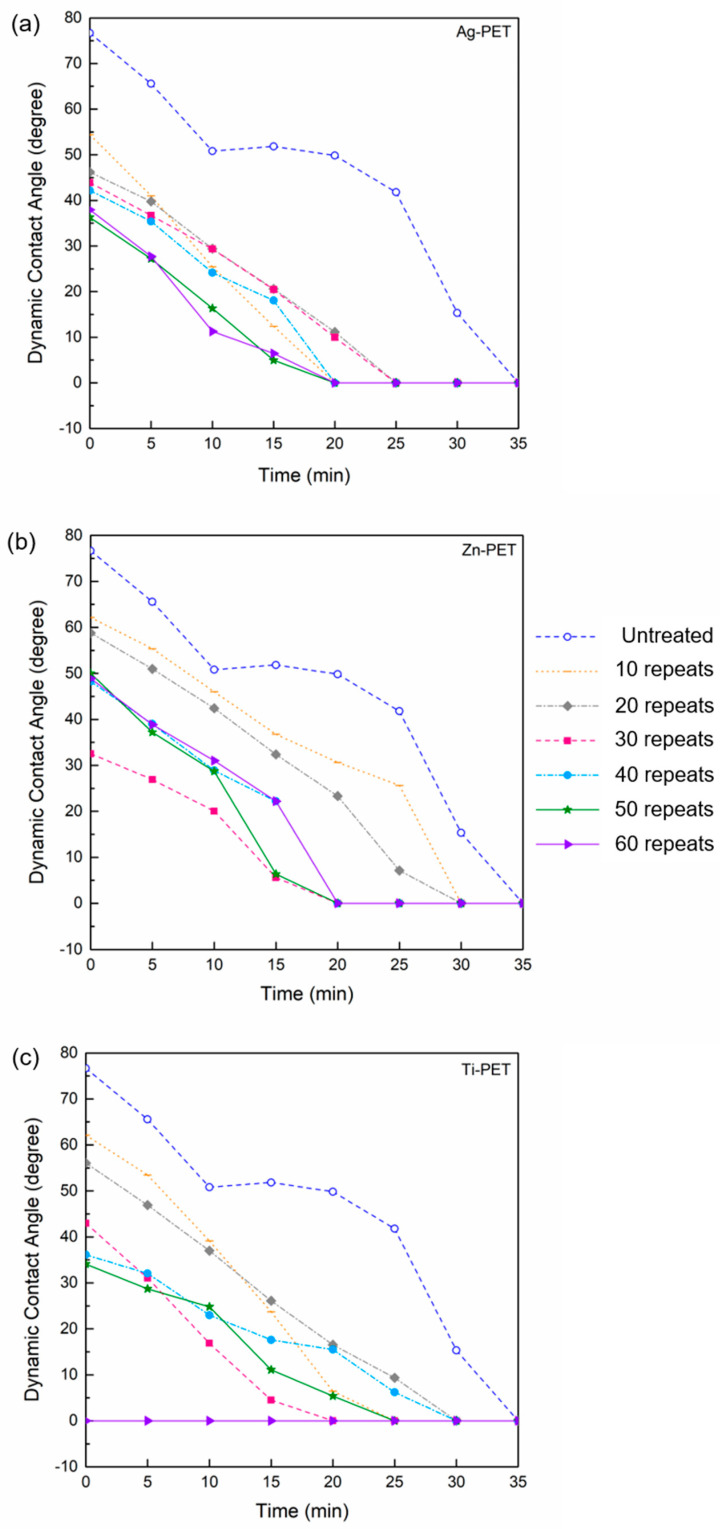
Dynamic contact angle curves of the (**a**) Ag-PET, (**b**) Zn-PET, and (**c**) Ti-PET films treated 0–60 times.

Figure 6 shows the dynamic water contact angle of the films. The untreated PET film showed a water contact angle of 78° at 0 min, and then continuously decreased to 0° at 35 min. The water contact angle of the treated PET films decreased with repeats and went to 0° within 15–30 min (60 repeats). The Ag-PET samples showed a reduction in the contact angle at 0 min (from 55 to 3° with 10–60 repeats) compared to untreated PET (78°), and rapidly decreased to 0° at 20–25 min. All metal ion treatments showed that the contact angle decreased with sparking times and testing time. The water contact angle of the Ti-PET samples significantly decreased with sparking times due to the high polarity and continuous aggregate morphology of the Ti phase (Figure 1b,c). The NP of metal ions probably decreased the water contact angle due to the reduction in interfacial tension because of the high polarity and continuous aggregate morphology of metal ions. The decrease in the contact angle and super-hydrophilic mechanism attributed to sparking a nano-metal ion on a polymer surface has been reported [39].

### 2.4. In Vivo and In Vitro Antivirus Property of the Nano-Metal Treated PET Films

**Figure 7:**
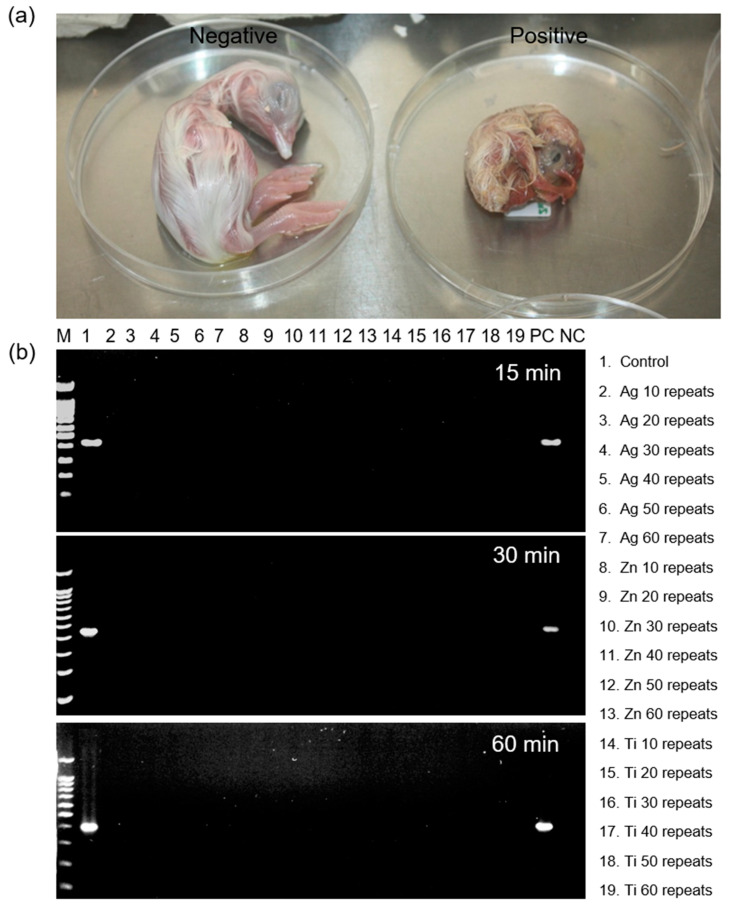
In vivo and in vitro anti-virus properties of the nano-metal-treated PET films: (**a**) Infected embryonic chick images of virus sensitivity showing signs of disease and (**b**) Infectious bronchitis virus amplification of the metal-nano treated PET films (60 repeats).

The surfaces of the untreated and treated PET films were treated with infectious bronchitis virus and inoculated into embryonic eggs. The infected embryonic eggs showed signs of disease or impending death (Figure 7a). The RT-PCR method was used to observe anti-virus properties on the surface of all sparked films. Redsafe^®^-stained agarose gels showed the results of infectious bronchitis virus amplification. The amplicon sizes are shown in the left margin (M). Results of the RT-PCR showed the 400 bp amplicon in lane 1 (untreated PET film), which was the PC (positive control). An additional negative control (NC) was included that contained 2 μL of the RT-PCR NC. Lanes 2–19 were the treated PET film samples with 10–60 repeats, which showed the lack of virus activity at 15, 30, and 60 min of virus incubation (Figure 7b), indicating that the metal NP on the surface of the PET films prevented coronavirus growth. This was probably due to their binding with the viral envelop protein of the viruses [22,23], photocatalytic oxidation [27,28], and physical damage [24,25,26] that destroyed the viral envelop protein of the viruses [19,20,41]. The PET treated with Zn (30 repeats) was selected to prepare a face shield as a representative application for the COVID-19 pandemic era because the Zn-PET (30 repeats) sample had a high transparency similar to an untreated PET sample, which would have no effect on visual performance of the films.

### 2.5. Face Shield Preparation and Observation of Properties

**Figure 8:**
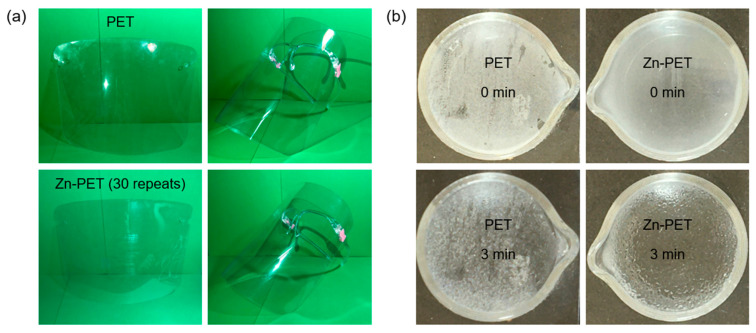
Images of the PET and Zn-PET (30 repeats) face shields (**a**) and images of water vapor fogging of the PET and Zn-PET films at 0 and 3 min (**b**).

**Figure 9:**
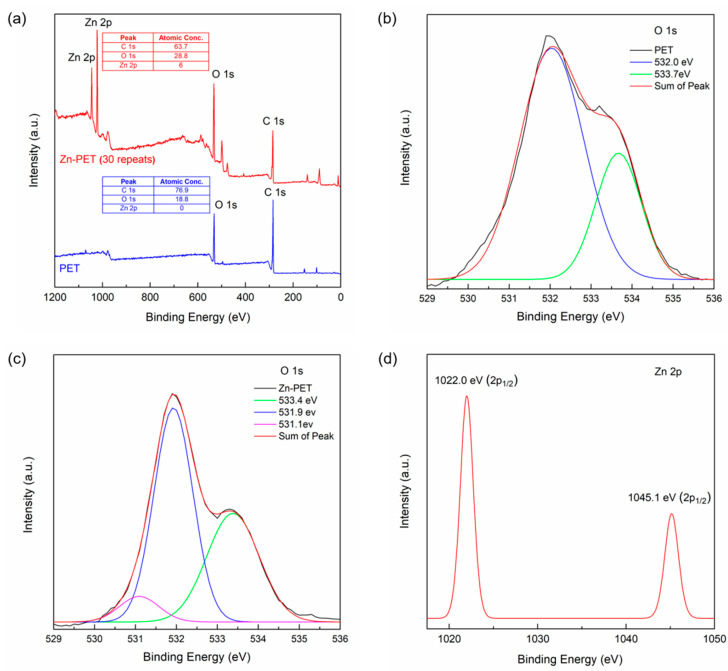
XPS survey spectra of PET and Zn-PET (30 repeats) (**a**), XPS spectra of O1s for PET (**b**) Zn-PET (30 repeats), and (**c**) films, and XPS spectra of Zn 2p_1/2_ and 2p_3/2_ (**d**).

An image of the face shield is shown in Figure 8a, and XPS was used to study the surface chemical structure. As shown in Figure 9a, the C:O atomic ratio of the untreated PET film was 76.9:18.8. After sparking with Zn for 30 repeats, the atomic concentration of C decreased from 76.9 to 63.7%, whereas the atomic concentration of O increased from 18.8 to 28.8%. The atomic concentration of Zn was 6.0%. Thus, the results confirmed that the ZnO NP was deposited on the PET film surface. As shown in Figure 9b, the untreated PET film showed oxygen peaks of C-O and C=O groups at 532.0 and 533.7 eV, respectively [48]. The Zn-PET film showed oxygen peaks of C-O and C=O groups similar to the untreated PET. However, the new binding energy peaks of Zn^2+^ ion were observed at 1022 and 1045 eV (Figure 9c,d). In addition, the Gaussian curve fitting of the Zn-PET film showed a new shoulder peak at 531.1 eV, which corresponds to the lattice oxygen of ZnO. These results are consistent with the previous study, which reported that the lattice oxygen of ZnO was present at low binding energies (i.e., 530.6, 530.2, 530.2, and 530.3 eV) [49,50,51]. However, the shoulder peak was observed at a higher energy of 531.1 eV compared to 530.6–530.2 eV because of ZnO NP embedded on the PET surface during the sparking process [52].

**Figure 10:**
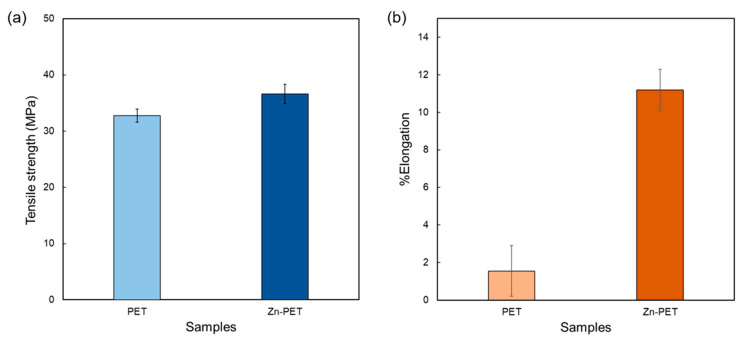
Tensile strength (**a**) and elongation at break (**b**) of the PET and Zn-PET (30 repeats) films. Means with different lowercase superscript letters are significantly different (*p* < 0.05).

When the film was placed above hot water steam, the untreated PET films were opaque with a high amount of fogging, while the treated films were fully transparent (Figure 8b). The tensile strength and elongation at the break of PET (32.7 MPa and 1.5%, respectively) were increased with the Zn NP sparking (36.6 MPa and 11.2%, respectively) as shown in Figure 10a,b. The mechanical properties and crystallinity improvements with NP modifications on polymer surfaces have been previously reported [53].

## 3. Materials and Methods

### 3.1. Materials

The PET film (0.25 mm thick) was purchased from the Janice Office Supplies Co., Ltd. (Bangkok, Thailand). The Ag, Zn, and Ti wires (0.5 mm diameter) were purchased from Advent Research Materials Co., Ltd. (Oxford, UK). Infectious bronchitis virus as a model coronavirus of chicken was supplied by the Veterinary Diagnostic Center, Faculty of Veterinary Medicine, Chiang Mai University, Chiang Mai, Thailand.

### 3.2. Sample Preparation

The two sharp tips (20 mm length) of metal wire were connected with the anode and cathode of a sparking machine. The tips were aligned at 2 mm above the PET film with a 1 mm gap between the anode and cathode. The in-house sparking system is shown in Figure 1a. PET film was sparked at 3 kV and 3 mA, and the tip holders of the wires were moved along the XY axes at a speed of 100 mm/min. The sparking process was repeated 10, 20, 30, 40, 50, and 60 times at 3 s/spark.

### 3.3. Morphology Analysis

Morphology of the samples was observed using an energy-dispersive X-ray spectroscope (EDS) (X-MaxN 20, Oxford Instrument Co., Ltd., Abingdon, UK) connected with an X-ray element mapping for the analysis of the surface atomic ratio, the elemental distribution, and a field-emission scanning electron microscope (FE-SEM) (JSM6335F, Oxford Instrument). The surface of the samples was coated with a thin layer of gold by a sputter coater (SPI-Module, 12157Q-AB, Structure Probe Co., Ltd., West Chester, PA, USA) and observed with an acceleration voltage of 10 kV. An atomic force microscope (AFM) (Nano Scope IIIa, Veeco Metrology Co., Ltd., Horsham, PA, USA) was used to observe the surface morphology of the PET film. The tapping mode used a resonant frequency of 200 kHz. The distribution curve and root mean square (rms) roughness values of the 3D images were generated using the Nanoscope III 5.12r3 software (Santa Barbara, CA, USA).

### 3.4. Color Measurement

The color of the untreated and treated PET samples was observed at the different repeat sparking times using a color meter (CR-10, Minolta, Tokyo, Japan). The L*, a*, and b* chroma system was used. The color difference (ΔE) was calculated using Equation (1):

ΔE = [(ΔL∗)^2^ + (Δa∗)^2^ + (Δb∗)^2^]^1/2^ (1)

where ΔL* is the brightness difference; Δa* is the redness-greenness difference; and Δb* is the yellowness-blueness difference between the treated and untreated films.

### 3.5. Dynamic Water Contact Angle Measurement

Drop shape analysis (DSA30E, Krüss Co., Ltd., Hamburg, Germany) was used to observe the water droplet contact angle. The water angle on the surface of the sample sheets was observed automatically every min until the angle was 0°.

### 3.6. Antivirus Test on the PET Film Surface

Three mL of the bronchitis-virus-infected allantoic fluid was prepared. Autoclaved cotton was swabbed with the virus solution and spread on the PET film surface and incubated at 25 °C for 15, 30, and 60 min. The virus sample was collected using a sterile cotton swab on the testing surface and put into 10X phosphate-buffered saline (PBS) containing 1.37 M sodium chloride, 27 mM potassium chloride, 100 mM sodium hydrogen phosphate, and 18 mM potassium phosphate, and chilled at 4 °C. Then, the allantoic sac of a 9 day-incubated specific-pathogen-free egg from a specific pathogen-free chicken farm (Cobb; *Gallus gallus domesticus*) (Betagro Co., Ltd., Bangkok, Thailand) was inoculated using the allantonic route with the supernatant of the sample in PBS. The supernatant was prepared by centrifuging the sample at 123 G force for 10 min. Then, 500 µL of supernatant was collected by using a micropipette (Eppendoff, Hamburg, Germany). After 48 to 72 h incubation at 37 °C, all inoculated embryonic eggs showing signs of disease or impending death were chilled to 4 °C before harvesting the allantoic fluid. A punch was used to open the egg at the original inoculation hole and the allantoic fluid was withdrawn by inserting a syringe needle. Allantoic fluid (10 mL) was kept at 4 °C prior to the PCR test.

RNA was extracted using the ReverTra Ace^®^ qPCR RT kit according to the manufacturer’s specifications (Toyobo Co., Ltd., Osaka, Japan). Then, cDNA amplification was conducted in a PCR thermal cycler (C100 TouchTM Thermal Cycler, Bio-Rad, Hercules, CA, USA), and reagents were from the Quick Taq HS DyeMix^®^ (Toyobo). Primers were Outer–F and Outer-R (Bio Basic Co., Ltd., Markham, ON, Canada) with target gene S1 [54] and are shown in detail in Table 2. Cycling conditions involved an initial 2 min denaturation at 95 °C, followed by 40 cycles, each consisting of a 30 s denaturation at 95 °C, a 30 s annealing at 52 °C, and a 30 s extension at 68 °C. These 40 cycles were followed by a 12 min extension at 68 °C and used as templates for nested reactions. Nested amplifications used 1 mL of the primary PCR product as the template in a total volume of 40 mL. Primers were Inner-F and Inner-R and are shown in detail in Table 2. Nested cycling conditions were as described for the primary amplification. Reaction products were subsequently maintained at 4 °C. A 5 μL aliquot of each reaction was loaded onto the 1.5% agarose gels (Agarose A, Bio Basic), and analyzed using agarose gels electrophoresis (PowerPac, BioRad) at 100 V for 30 min. The 100 bp ladder (SibEnzyme, Novosibirsk, Russia) was used as the molecular weight marker. The study was carried out in accordance with the international ethical guidelines for animal experimentation, and all experiments were approved by the Institutional Animal Care and Use Committee, Faculty of Veterinary Medicine, Chiang Mai University, Chiang Mai, Thailand (permission number: FVM-ACUC.R15/2564).

### 3.7. Surface Analysis

X-ray photoelectron spectroscopy (XPS) (AXIS Ultra DLD, Kratos Analytical Co., Ltd., Manchester, UK) was used to observe the surface chemistry of the PET films. XPS spectra were corrected using a monochromatic Al Kα radiation, pass energy of 20 eV, and step size of 100 meV.

### 3.8. Anti-Fogging Analysis

The anti-fogging analysis of the film surface was measured using films placed above a 5 mL beaker containing 80 °C hot water (3 mL) for 3 min. The surface changes of the films were observed [55].

### 3.9. Tensile Test

Five specimens of the samples were observed using tensile testing (model H1KS, Hounsfield Test Equipment, Redhill, UK). The size of the samples was 5 × 30 × 0.25 mm. The samples were conditioned at 25 °C and 54% RH for 24 h. The samples were observed with a gap length of 10 mm with a test speed of 2 mm/min. Tensile strength and percentage elongation were measured at the maximum strength and percentage elongation at the breakpoint of the samples, respectively.

### 3.10. Statistical Analysis

All results were analyzed using one-way ANOVA with the Statistical Package for the Social Sciences software (Version 22, SPSS, Chicago, IL, USA). The differences found (*p* < 0.05) were evaluated using the Tukey test.

## 4. Conclusions

Owing to a high rate of infection of COVID-19, a high transparency of anti-coronavirus and anti-fogging film was developed using the sparking technique, depositing Ag, Zn, and Ti NP on PET films. The initial test of anti-coronaviral activity for the treated metal-PET films was conducted using bronchitis virus as a model coronavirus because it is safe in the laboratory. The morphology studies showed that Ag had color degradation while Zn and Ti aggregates were formed after a number of repeat sparking times. The water contact angle of the treated PET surface showed a super-hydrophilic surface. In vivo and in vitro anti-virus observations showed the significant anti-coronavirus properties of the treated metal-PET films. This research showed the effectiveness of NP deposition on a PET surface for an alternative PPE, which could be applied for COVID-19 pandemic protection. The deposition of NP on PET using a sparking technique may provide a workable technology for the mass production of PPE and for other applications.

## Figures and Tables

**Table 1:** Roughness of the Ag-PET, Zn-PET, and Ti-PET films treated 0–60 times.

Samples	Sparking Times	Roughness (nm)
Untreated PET	0	0.00
Ag-PET	10	3.58
	20	4.88
	30	7.25
	40	8.02
	50	9.42
	60	10.20
Zn-PET	10	15.95
	20	20.84
	30	29.48
	40	32.17
	50	36.87
	60	40.41
Ti-PET	10	6.35
	20	9.62
	30	11.69
	40	14.89
	50	18.51
	60	19.60

**Table 2:** PCR primers and PCR products.

Primer Name	Primer Sequence 5′–3′	Target Gene	Product Length
Outer-F Outer-R	CTT-TTG-TTT-GCA-CTA-TGT-AG TAA-TAA-CCA-CTC-TGA-GCT-GT	S1	878 bp
Inner-F Inner-R	CAG-TGT-TTG-TCA-CAC-ATT-GT CCA-TCT-GAA-AAA-TTG-CCA-GT	S1	400 bp

## Data Availability

Correspondence and requests for materials should be addressed to P.R.
